# Comparative Genomics Revealed Multiple *Helicobacter pylori* Genes Associated with Biofilm Formation *In Vitro*

**DOI:** 10.1371/journal.pone.0166835

**Published:** 2016-11-21

**Authors:** Eric Hong Jian Wong, Chow Goon Ng, Eng Guan Chua, Alfred Chin Yen Tay, Fanny Peters, Barry J. Marshall, Bow Ho, Khean Lee Goh, Jamuna Vadivelu, Mun Fai Loke

**Affiliations:** 1 Department of Medical Microbiology, Faculty of Medicine, University of Malaya, Kuala Lumpur, Malaysia; 2 Department of Microbiology, Yong Loo Lin School of Medicine, National University of Singapore, Singapore, Singapore; 3 The Marshall Centre for Infectious Diseases Research and Training, School of Pathology and Laboratory Medicine (M502), University of Western Australia, Perth, Australia; 4 UM Marshall Centre, High Impact Research Building, University of Malaya, Kuala Lumpur, Malaysia; 5 Department of Medicine, Faculty of Medicine, University of Malaya, Kuala Lumpur, Malaysia; Monash University, AUSTRALIA

## Abstract

**Background:**

Biofilm formation by *Helicobacter pylori* may be one of the factors influencing eradication outcome. However, genetic differences between good and poor biofilm forming strains have not been studied.

**Materials and Methods:**

Biofilm yield of 32 *Helicobacter pylori* strains (standard strain and 31 clinical strains) were determined by crystal-violet assay and grouped into poor, moderate and good biofilm forming groups. Whole genome sequencing of these 32 clinical strains was performed on the Illumina MiSeq platform. Annotation and comparison of the differences between the genomic sequences were carried out using RAST (Rapid Annotation using Subsystem Technology) and SEED viewer. Genes identified were confirmed using PCR.

**Results:**

Genes identified to be associated with biofilm formation in *H*. *pylori* includes alpha (1,3)-fucosyltransferase, flagellar protein, 3 hypothetical proteins, outer membrane protein and a *cag* pathogenicity island protein. These genes play a role in bacterial motility, lipopolysaccharide (LPS) synthesis, Lewis antigen synthesis, adhesion and/or the type-IV secretion system (T4SS). Deletion of *cagA* and *cag*PAI confirmed that CagA and T4SS were involved in *H*. *pylori* biofilm formation.

**Conclusions:**

Results from this study suggest that biofilm formation in *H*. *pylori* might be genetically determined and might be influenced by multiple genes. Good, moderate and poor biofilm forming strain might differ during the initiation of biofilm formation.

## Introduction

*Helicobacter pylori* is strongly associated with gastroduodenal diseases, such as chronic gastritis, peptic ulcer, duodenal ulcer and gastric cancer [1]. In fact, this bacteria has been classified as a group I carcinogen by the International Agency for Research on Cancer [2]. Although *H*. *pylori* is susceptible to most antibiotics *in vitro*, only few antibiotics can be used for eradicating *H*. *pylori in vivo*, e.g. amoxicillin, clarithromycin, metronidazole and tetracycline [3]. Combination therapy is needed for successful eradication of *H*. *pylori*, Increasing prevalence of antibiotic resistance in *H*. *pylori* is problematic since it is one of the important causes of therapy failure [4]. The ability of *H*. *pylori* to form biofilm *in vitro* and also *in vivo* has been demonstrated [5–7]. *H*. *pylori* formed biofilm within *in vivo* environment especially gastric mucosa had been well demonstrated by previous study [7]. The formation of biofilm within *in vivo* environment was believed to be one of the factors which cause *H*. *pylori* eradication failure [7].

According to Donlan *et al*. (2002) [8], biofilms are defined as complex microbial ecosystems adherent to each other and/or to surface or interface. It is a microbially derived sessile community characterized by cells that are embedded in a matrix of extracellular polymeric substances that they have produced, and exhibit an altered phenotype with respect to growth rate and gene transcription [8]. Biofilms are important in bacterial pathogenesis as they plays a central role in helping microbes survive or spread within the host. This is because the biofilm matrix acts as shield, protecting bacteria from host defenses and antibiotics [9,10]. Bacterial biofilm can be 10 to 1000 times less susceptible to antimicrobial substances than the same organism in suspension [11]. Biofilm formation makes it difficult for antibiotics to reach the bacteria, underneath aiding the development of antibiotic resistance and increase the risk of treatment failure [8]. Therefore, biofilms are often associated with chronic infections [12]. Cammarota *et al*. demonstrated that *H*. *pylori* biofilm on the gastric mucosa epithelium was associated with eradication failure and N-acetylcysteine pretreatment before antibiotic therapy was effective in overcoming antibiotic resistance [13,14].

The ability of biofilm formation in bacteria is genetically controlled [15–19]. Based on previous studies, one of the most common genes associated with biofilm formation in bacteria (including *H*. *pylori*, *S*. *mutans* and *L*. *monocytogenes*) is the *luxS* gene [15–17]. Apart from *luxS* gene, few other genes have also been reported to be associated with biofilm formation in different bacteria [18,19]. According to Yoshida and Kuramitsu (2002) [20], *dgk*, *sgp* and *com* also play a role in *S*. *mutans* biofilm formation. Wu and colleagues [21] revealed that *treC* and *sugE* affect biofilm formation in *K*. *pneumoniae* by modulating capsular polysaccharide synthesis production. Taken together, biofilm formation in bacteria may be regulated by multiple genes working together.

Despite extensive studies conducted on *H*. *pylori* biofilm formation, precisely how genetic differences of different *H*. *pylori* strains influence biofilm formation remains not well-established. The objective of this study is to identify genes associated with *H*. *pylori* biofilm formation.

## Materials and Methods

### *H. pylori* strains

*H*. *pylori* standard strain J99 (ATCC 700824) was obtained from the American Type Culture Collection (USA). The *H*. *pylori* clinical strains used in this study were from the *H*. *pylori* collection of the *Helicobacter* Research Laboratory at the University of Malaya (Malaysia).

*H*. *pylori* strain 26695 (ATCC 70392) *cagA* and *cag*PAI deletion mutants were constructed using the PCR-based method as described by Chalker *et al*. [22] and Tan and Berg (2004) [23] in A/Prof. Ho Bow’s laboratory at the Department of Microbiology (National University of Singapore, Singapore).

### Culturing of the *H*. *pylori* and biofilm

*H*. *pylori* were cultured on non-selective chocolate blood agar plate and incubated in a humidified incubator with 10% CO2 at 37°C for three days. The 3-days old *H*. *pylori* plate cultures were harvested with a sterile cotton swab and emulsified in brain heart infusion (BHI) broth (Oxoid, Hampshire, UK) supplemented with 1% β-cyclodextrin (Sigma-Aldrich, St. Louis, MO, USA) and 0.4% yeast extract (Oxoid, Hampshire, UK). After 3 days incubation, the total bacterial population of each of the cultures was standardized with the same bacterial turbidity value (bacterial turbidity = 0.042). Following which, 500 μl of bacterial suspension was then inoculated into each well of a 24-well tissue culture plate (BD Falcon, Franklin Lakes, NJ, USA). BHI broth without *H*. *pylori* served as a negative control. The growth of the biofilm was observed at the day 3, day 5, day 7 and day 14 with the amount of biofilm formed analyzed based on crystal violet staining.

At each time interval, the bacterial suspensions of the 24-well tissue culture plate were first aspirated out. An aliquot of 650μl of 0.1% crystal violet (Friendemann Schmidt, WA, Australia) was added to each well and the plate was gently swirled for 30 minutes to ensure uniform staining of the biofilm. The excess crystal violet stain was removed and the wells were washed thoroughly using 800μl sterile distilled water to remove any excess crystal violet stain. The crystal-violet stained biofilm was finally destained using 1ml of 95% ethanol (VWR Prolabo, Lutterworth)-5% acetic acid (Friendemann Schmidt, WA, Australia) which serve as destaining solution. The solution collected was measured at OD_600nm_ on a SmartSpec Plus spectrophotometer (Bio-Rad, Hercules, CA, USA). The amount of crystal violet absorbed by the biofilm (CV_600nm_) was determined by taking the mean absorbance value. All experiments were performed technical triplicates and in difference day of biological triplicates.

At the same time, bacterial turbidity value of each bacteria strains were taken for every time points. The bacterial turbidity value of each strain was taken in order to determine the growth rate of the strains. All experiments were performed in biological and technical triplicates and specific biofilm unit (CV_600nm_ /OD_600nm_) value was calculated.

### Illumina Library Preparation and Sequencing

DNA was extracted from 3-day old *H*. *pylori* using the Bacterial DNA mini kit (Stratec Molecular, Berlin, Germany) according to the manufacturer’s instructions. Illumina sequencing library was prepared and samples were sequenced on a MiSeq sequencer (Illumina, San Diego, CA, USA) as described in Perkins *et al*. [24]. Sequences used in this study have been deposited with GenBank under accession numbers listed in **Table 1**.

**Table 1:** Accession numbers and details of *H*. *pylori* genomic sequences used in this study.

Strain	Accession no.	Genome size (bp)	Genes	Proteins	%GC	Contigs	Max length (bp)	N50 (bp)	Coverage	Ref
J99	NC_000921	1,643,831	1,559	1,471	39.2	1	1,643,831	1,643,831	-	Alm *et al*, 1999 [25]
UM032	NC_021215	1,593,537	1549	1438	38.8	1	1,593,537	1,593,537	-	Yalda *et al*., 2013 [26]
UM023	AUSK00000000	1,624,154	1,562	1,518	38.7	34	485,260	183,178	-	Rehvathy *et al*., 2013 [27]
UM037	AUSI00000000	1,724,611	1,645	1,574	38.9	60	234,132	80,609	-	Rehvathy *et al*., 2013
UM065	AUSM00000000	1,587,249	1,500	1,461	38.9	39	334,064	163,534	-	Rehvathy *et al*., 2013
UM066	AUSJ00000000	1,694,309	1,590	1,562	38.6	34	319,894	146,858	-	Rehvathy *et al*., 2013
UM077	AUSQ00000000	1,620,877	1,565	1,527	38.8	53	328,671	187,040	-	Rehvathy *et al*., 2013
UM085	AUSP00000000	1,645,640	1,568	1,524	38.7	50	341,517	94,888	-	Rehvathy *et al*., 2013
UM111	AUSR00000000	1,663,383	1,581	1,536	38.7	38	245,602	110,134	-	Rehvathy *et al*., 2013
UM045	AONO00000000	1,602,114	1,559	1,439	39.0	20	110,455	146,660	158	This study
UM054	AONL00000000	1,594,474	1,570	1,422	39.1	81	103,788	39,593	148	This study
UM087	LFDR00000000	1,657,861	1,610	1,456	38.9	17	260,866	153,641	166	This study
UM119	LFBY00000000	1,592,071	1,564	1,439	38.8	18	266,685	153,847	153	This study
UM122	LFBX00000000	1,593,819	1,556	1,427	39.1	23	346,159	96,269	124	This study
UM131	LFBZ00000000	1,598,266	1,551	1,422	39.2	16	375,408	158,380	151	This study
UM139	LFCA00000000	1,617,602	1,574	1,454	38.9	20	231,133	90,197	180	This study
UM147	LFLE00000000	1,633,271	1,567	1,442	39.5	58	272,592	145,059	153	This study
UM152	LFIS00000000	1,633,599	1,568	1,445	39.0	16	434,701	188,275	169	This study
UM158	LFCB00000000	1,634,841	1,588	1,448	38.9	27	240,532	185,427	153	This study
UM163	LFJR00000000	1,656,598	1,601	1,475	38.5	16	314,921	140,690	175	This study
UM165	LFIR00000000	1,651,447	1,611	1,471	38.9	20	414,265	125,433	116	This study
UM202	LFKE00000000	1,672,999	1,664	1,494	38.9	62	117,987	40,452	214	This study
UM246	LFKI00000000	1,644,161	1,592	1,466	38.6	16	277,351	174,853	200	This study
UM276	LJXI00000000	1,666,681	1,689	1,512	38.8	61	330,937	158,795	157	This study
UM291	LFKJ00000000	1,569,053	1,547	1,418	38.8	28	219,046	123,332	135	This study
UM300	LFIT00000000	1,591,764	1,535	1,410	39.2	14	259,105	193,002	142	This study
UM352	LFKK00000000	1,623,273	1,573	1,443	38.7	19	330,865	156,967	202	This study
UM370	LFKL00000000	1,582,384	1,552	1,422	38.7	23	200,250	93,564	174	This study
UM408	LFIU00000000	1,576,513	1,524	1,391	39.1	26	265,219	111,980	253	This study
UM411	LFKM00000000	1,631,715	1,582	1,440	39.0	17	271,501	151,419	201	This study
UM520	LEOV00000000	1,617,859	1,605	1,444	38.7	51	177,886	52,801	160	This study

### Identifying genes associated with biofilm formation

Biofilm forming ability of *H*. *pylori* standard strains J99 and 31 sequenced clinical strains were determined. Based on highest biofilm specific unit value, there strains were classified into 3 groups (good, moderate and poor biofilm formers. Cumulative frequency (%) was calculated using the formula:

$$cumulative\;frequency\;(\%)for\;32\;strains=a+\left(\frac{x}{32}\times 100\right)$$

Where,

a = previous cumulative frequency;

x = number of strains which showed the particular highest biofilm specific unit value

The rate of biofilm formation for each of the strains was determined by using the formula below:

$$Rate\;of\;biofilm\;formation=\frac{biofilm\;formation\;day\;b-biofilm\;formation\;day\;a}{day\;b-day\;a}$$

*H*. *pylori* genomic sequences were annotated and compared using RAST (Rapid Annotation using Subsystem Technology) (http://rast.nmpdr.org/) [28–30]. Sequence-based comparison among *H*. *pylori* strains was carried out using the SEED viewer of RAST with percentage similarity above 80% was used. Two-tailed Fisher’s exact test was carried out using SPSS (IBM statistic 20, Armonk, New York, USA). P-value <0.05 was considered statistically significant.

Next, the presence and absence of genes-of-interest identified by comparative genomic analysis were confirmed by conventional end-point PCR using primers and conditions listed in **Table 2**.

**Table 2:** Primers used for amplification of *H*. *pylori* biofilm genes.

Gene	Primer sequence (5’- 3’)	Product size (bp)	PCR condition
hypothetical protein K747_10375	Forward: CATCTCGCGTGATGGGGT	426	95°C, 5min; 35x (95°C, 30s; 53°C, 30s; 72°C, 1min); 72°C, 7min
	Reverse: TCTTCTTGCTTTTTGGCGAT		
hypothetical protein K747_09130	Forward: GAGTGGGATAGAGTTAGAAC	777	95°C, 5min; 35x (95°C, 30s; 60°C, 30s; 72°C, 1min); 72°C, 7min
	Reverse: GTATTAGCCGCTGCTTC		
flagellar protein	Forward: GTGAGTTGTGCATACGCT	388	95°C, 5min; 35x (95°C, 30s; 51°C, 30s; 72°C, 1min); 72°C, 7min
	Reverse: AGGCCACTGAGTTTTTAGGT		
alpha-(1,3)-fucosyltransferase	Forward: TCCAGCCCTTACTAGACGCT	1279	95°C, 5min; 35x (95°C, 30s; 57°C, 30s; 72°C, 1min); 72°C, 7min
	Reverse: AGCTCCAAAAGAGGGGTAGC		
hypothetical protein K747_06625	Forward: GGCTCACCACTATACCGCTT	1089	95°C, 5min; 35x (95°C, 30s; 57°C, 30s; 72°C, 1min); 72°C, 7min
	Reverse: TGACCGGCTCTTTTGTGTCA		
outer membrane protein (*homD*)	Forward: GACGCTCAAGGCAAGGTAGT	1409	95°C, 5min; 35x (95°C, 30s; 57°C, 30s; 72°C, 1min); 72°C, 7min
	Reverse: AACACATCCATTCCCCCACC		
*cag* pathogenicity island protein	Forward: AACGCTCCATCAAGAGCCAA	1332	95°C, 5min; 35x (95°C, 30s; 57°C, 30s; 72°C, 1min); 72°C, 7min
	Reverse: CCCGCTCTTGCTTCCTTACT		

### Scanning electron microscopy (SEM)

**Fig 1:**
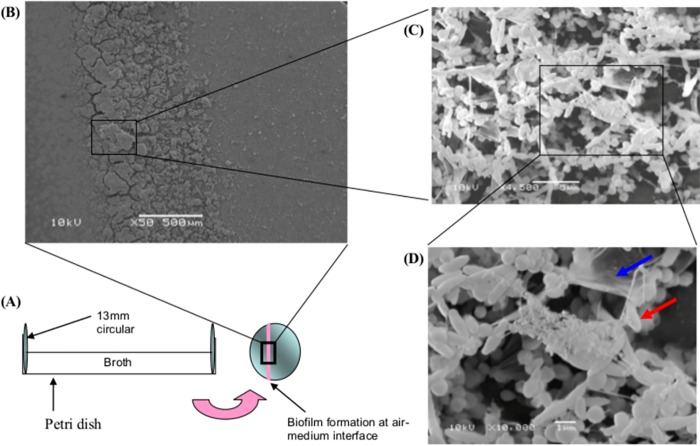
SEM of *H*. *pylori* biofilm strain 26695 on coverslip. (A) Diagrammatic illustration of *H*. *pylori* biofilm formation on coverslip. (B) Section of *H*. *pylori* at the air medium interface, showing biofilm formation on day 14 (x50). (C) Enlarged section of *H*. *pylori* biofilm formed at the air medium interface (x4,500). (D) Enlarged section of *H*. *pylori* biofilm showing the presence of film like structures (x10,000). Blue arrow indicate the film-like structures postulated to be exopolysaccharides and red arrow indicate spiral *H*. *pylori*.

Scanning Electron Microscopy (SEM) was performed as described by Lemos et al. (2004) with modifications [31]. The biofilms were grown on the coverslips (**Fig 1**). The coverslips were washed 2 times in sterile phosphate buffered saline (PBS). The coverslips were then fixed with 2% (wt/vol) glutaraldehyde in 0.1 M PBS (pH 7.4) and post-fixed with 1% osmium tetroxide in 0.1M phosphate buffered saline. The fixed cells were washed with 0.1 M PBS and dehydrated with 50%, 75%, 95%, 100% ethanol. The samples were dried using critical point dryer (Bal-Tec CPD 0300) and examined using JSM-5600 scanning electron microscope (JEOL, Peabody, MA, USA) operating at 10kV.

## Results

### Optimization of conditions for *in vitro* biofilm formation

*H*. *pylori* biofilm formation was observable at the air-liquid interface of wells after three days of incubation in a humidified 10% CO2 incubator at 37°C. Optimum conditions for biofilm formation were chosen based on maximum biofilm formation without compromising bacterial growth. Comparing between different concentrations of β-cyclodextrin (0.5% to 2.0%) and fetal bovine serum (5% to 15%) as supplement to BHI broth, 1% β-cyclodextrin was shown to be optimum for biofilm formation. Biofilm formation in BHI with 1% β-cyclodextrin broth showed at least one-fold higher than BHI supplemented with fetal bovine serum (5% to 15%) and β-cyclodextrin (0.5% and 2.0%). Biofilm formation in microaerophilic condition was shown to be 3-fold higher than ambient atmospheric condition. As osmolality of the medium was increased (from original 0.5% to 1% and 2% sodium chloride), biofilm formation and specific biofilm unit were reduced. In addition, the optimum condition for both biofilm formation and growth occurred at pH 7.0. Thus, BHI broth supplemented with 0.4% yeast extract and 1% β-cyclodextrin at pH 7.0 and microaerophilic condition was determined to be optimum for the study of biofilm formation of *H*. *pylori* in this study.

### Identification of genes associated with biofilm formation

The median biofilm specific unit value (cumulative frequency = 50%) was determined to be between specific biofilm unit of 1.0 and 1.1. Strains producing biofilm with specific biofilm unit <1.0 were classified as poor biofilm formers while those >1.1 were classified as good biofilm formers. Those specific biofilm units between 1.0 and 1.1 were classified as moderate biofilm formers. Twelve (37.5%) of the tested strains were classified as good biofilm formers, while another 12 (37.5%) strains were poor biofilm formers. The remaining 8 (25.0%) strains were moderate biofilm formers (**Table 3**). Highest specific biofilm unit was shown to be significantly different between poor, moderate and good biofilm formers (one-way ANNOVA p-value = 0.001).

**Table 3:** Classification of *H*. *pylori* strains based on highest specific biofilm unit. One-way ANOVA was used to demonstrate statistically significant differences in highest specific biofilm unit between poor, moderate and good biofilm formers (p-value = 0.001).

Strain	Highest specific biofilm unit (CV_600nm_ /OD_600nm_)	Cumulative frequency (%)	BF Group
UM045	0.6	3.2	Poor
UM131	0.7	9.5	Poor
UM411	0.7	9.5	Poor
UM147	0.8	15.7	Poor
UM152	0.8	15.7	Poor
UM054	0.9	25.1	Poor
UM037	0.9	25.1	Poor
UM300	0.9	25.1	Poor
UM202	1.0	40.7	Moderate
UM065	1.0	40.7	Moderate
UM158	1.0	40.7	Moderate
UM276	1.0	40.7	Moderate
UM408	1.0	40.7	Moderate
UM023	1.1	62.6	Moderate
UM165	1.1	62.6	Moderate
UM520	1.1	62.6	Moderate
UM246	1.1	62.6	Moderate
UM370	1.1	62.6	Moderate
UM291	1.1	62.6	Moderate
UM139	1.1	62.6	Moderate
UM352	1.3	72.0	Good
UM122	1.3	72.0	Good
UM032	1.4	75.1	Good
UM087	1.5	81.4	Good
J99	1.5	81.4	Good
UM163	1.6	84.5	Good
UM119	1.7	87.6	Good
UM085	2.4	90.7	Good
UM077	4.0	93.9	Good
UM066	4.9	97.0	Good
UM111	5.0	100.0	Good

Median highest specific biofilm unit between 1.0 and 1.1 was classified as moderate biofilm formers. Highest specific biofilm unit shown represent average of triplicates. BF: biofilm former

Based on rate of biofilm formation from day 0 to day 7, there was clear segregation between poor and good biofilm forming strains (**Table 4**). Student’s t-test revealed that there were significant differences in rate of biofilm formation between poor, moderate and good biofilm forming strains across all time point (p-value <0.05), except between poor and moderate groups at day 3 to day 5 (p-value = 0.260). While good and poor biofilm forming strains displayed relatively consistent rate of biofilm formation across 7 days, moderate biofilm forming strains varied in rate of biofilm formation across time course of study. The moderate biofilm forming strains showed either declined biofilm forming rate by day 3 or delayed optimum biofilm formation starting from day 3 onwards. The rate of biofilm formation profiles was consistent with the highest specific biofilm unit profiles across multiple *H*. *pylori* strains.

**Table 4:** The rate of biofilm formation for all the *H*. *pylori* strains which were used in this study. Statistical significance for differences in rate of biofilm formation across time points was determined by two-tailed Student’s t-test.

Strain	BF Group	Rate of biofilm formation (biofilm formation per day)		
		Day 0 to 3	Day 3 to 5	Day 5 to 7
UM045	Poor	0.012	0.023	0.010
UM131	Poor	0.018	0.012	0.022
UM411	Poor	0.021	0.022	0.019
UM147	Poor	0.018	0.108	0.032
UM152	Poor	0.016	0.035	0.024
UM054	Poor	0.013	0.112	0.033
UM037	Poor	0.012	0.011	0.011
UM300	Poor	0.015	0.011	0.011
UM202	Moderate	0.025	0.035	0.128
UM065	Moderate	0.100	0.059	0.034
UM158	Moderate	0.100	0.132	0.066
UM276	Moderate	0.027	0.110	0.092
UM408	Moderate	0.080	0.041	0.021
UM023	Moderate	0.019	0.165	0.147
UM165	Moderate	0.022	0.056	0.067
UM520	Moderate	0.030	0.055	0.026
UM246	Moderate	0.032	0.033	0.105
UM370	Moderate	0.103	0.029	0.021
UM291	Good	0.039	0.039	0.125
UM139	Good	0.018	0.028	0.093
UM352	Good	0.091	0.137	0.100
UM122	Good	0.127	0.128	0.128
UM032	Good	0.134	0.112	0.124
UM087	Good	0.136	0.146	0.151
J99	Good	0.133	0.133	0.157
UM163	Good	0.112	0.100	0.119
UM119	Good	0.103	0.112	0.099
UM085	Good	0.123	0.120	0.089
UM077	Good	0.108	0.113	0.118
UM066	Good	0.153	0.148	0.136
UM111	Good	0.162	0.158	0.151
**Average**				
*Poor BF*	-	0.016	0.042	0.020
*Moderate BF*	-	0.050	0.065	0.077
*Good BF*	-	0.126	0.128	0.125
**Student’s t-test**				
*Poor vs*. *Moderate BF*	-	0.006*	0.260	0.001*
*Moderate vs*. *Good BF*	-	<0.001*	0.001*	0.005*
*Poor vs*. *Good BF*	-	<0.001*	<0.001*	<0.001*

Rate of biofilm formation shown represents average of triplicates; BF: biofilm former

* p-value<0.05 is considered statistically significant

**Fig 2:**
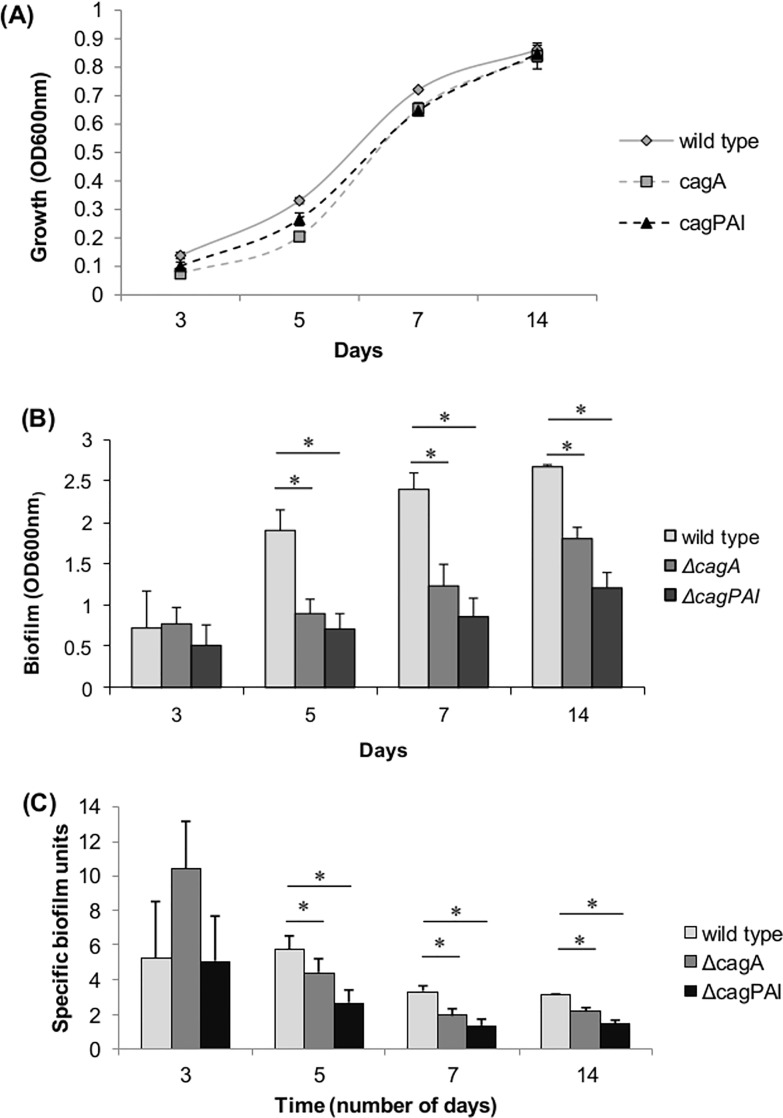
Bacterial growth and biofilm formation by *H*. *pylori* 26695 wild-type, *cag*A and *cag*PAI deletion mutants. (A) Bacterial growth measured at OD600nm, (B) biofilm formation analyzed using crystal violet analysis and (C) specific biofilm unit. Specific biofilm unit is a representation of biofilm formation normalized with planktonic mass and is determined by dividing the absorbance of biofilm-bound crystal violet with the corresponding planktonic cell turbidity OD. Biofilm development of different *H*. *pylori* strains was monitored over a period of 14 days. Values and error bars represent means and standard deviations of measurements in triplicates. (*) p< 0.05 (2-tailed Student’s *t* test).

**Fig 3:**
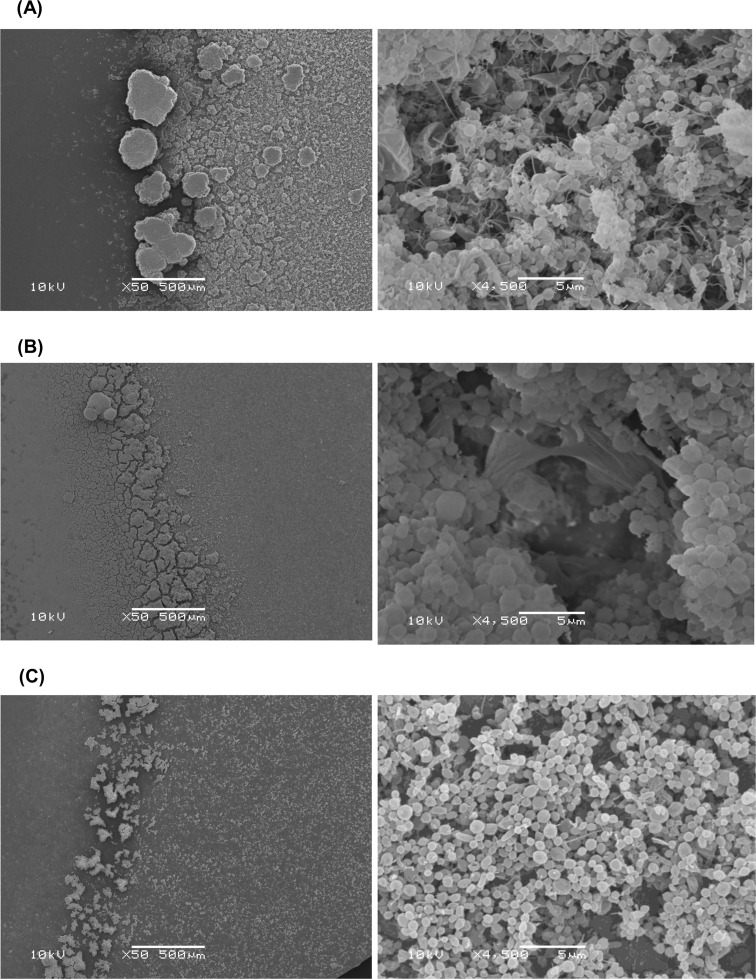
SEM of biofilm formed by 14-day old *H*. *pylori* 26695 wild-type, *cag*A and *cag*PAI deletion mutants. Biofilm formed by (A) wild type, (B) *cag*A and (C) *cag*PAI knockouts as observed under x50 and x4,500 magnification. The microcolonies in the biofilm were denser and bigger in wild type as compared to Δ*cag*A and Δ*cag*PAI. *cag*PAI knockout mutants formed sparsely distributed microcolonies at the air-medium interface.

Through the comparison of annotated genes present in *H*. *pylori* genomic sequences of poor, moderate and good biofilm forming strains, with 3 hypothetical genes (Hypothetical protein K747_10375, Hypothetical protein K747_09130 and Hypothetical protein K747_06625) and 4 functional genes (flagellar protein, *cag* pathogenicity island protein, outer membrane protein (*homD*) and alpha-(1,3)- fucosyltransferase) were identified to be significantly correlating with capability of forming biofilm (p-value <0.05) (**Table 5**). The presences and absences of these interested genes were verified by using PCR method (**Table 5**). One of the genes identified is annotated as *cag* pathogenicity island protein. To confirm that *cag* pathogenicity island (*cag*PAI) genes were involved in *H*. *pylori* biofilm formation, biofilm formation by wild-type and deletion mutants of *cagA* and *cag*PAI were compared. Bacteria turbidity and biofilm formation were increasing day 3 to day 14 (**Fig 2**). This was consistent with observation by SEM that spiral form of *H*. *pylori* could still found in the biofilm on day 14. Deletion of *cagA* and *cag*PAI were demonstrated to reduce biofilm formation (**Figs 2** and **3**). The microcolonies in the biofilm were denser and bigger in wild type as compared to Δ*cag*A and Δ*cag*PAI. *cag*PAI knockout mutants formed sparsely distributed microcolonies at the air-medium interface (**Fig 3**). At higher magnification, it was observed that the extracellular matrix formed by the wild-type strain was denser than that of Δ*cag*A and Δ*cag*PAI. In addition, extracellular matrix formed by Δ*cag*A was film-like in structure compared to the filament-like structure form by wild-type. Extracellular matrix was scanty in the biofilm of Δ*cag*PAI.

**Table 5:** Genes with significant association with biofilm formation (p-value <0.05) confirmed by PCR.

Genes	RefSeq reference no.	*Helicobacter pylori* strain (N = 32)			Fisher’s exact test (p-value)
		Poor biofilm former (N = 8)	Moderate biofilm former (N = 12)	Good biofilm former (N = 12)	
hypothetical protein K747_10375	WP_015645358.1	0	7 (58.3%)	12 (100%)	<0.001*
			UM065, UM276, UM023, UM520, UM246, UM370, UM291	UM122, UM032, UM087, J99, UM163, UM119, UM085, UM077, UM066, UM111, UM352	
hypothetical protein K747_09130	WP_015645548.1	1 (12.5%)	7 (58.3%)	11 (91.7%)	0.001*
		UM045	UM202, UM276, UM408, UM520, UM246, UM370, UM291	UM122, UM032, UM087, J99, UM163, UM119, UM085, UM077, UM066, UM111	
hypothetical protein K747_06625	WP_015644951.1	1 (12.5%)	8 (66.7%)	11 (91.7%)	<0.001*
		UM045	UM202, UM158, UM408, UM246, UM023, UM520, UM139, UM291	UM122, UM032, UM087, J99, UM163, UM119, UM085, UM077, UM066, UM352	
flagellar protein	WP_000744159.1	1 (12.5%)	8 (66.7%)	11 (91.7%)	<0.001*
		UM045	UM202, UM065, UM158, UM408, UM023, UM165, UM370, UM139	UM122, UM032, UM087, J99, UM163, UM119, UM085, UM077, UM066, UM352	
*cag* pathogenicity island protein	WP_015644978.1	0	7 (58.3%)	8 (66.7%)	0.005*
			UM065, UM276, UM023, UM520, UM246, UM370, UM291	UM032, UM163, UM119, UM085, UM077, UM066, UM352	
outer membrane protein (*homD*)	WP_002205258.1	0	7 (58.3%)	10 (83.3%)	0.001*
			UM065, UM276, UM408, UM520, UM370, UM291, UM139	UM122, UM087, J99, UM163, UM119, UM085, UM077, UM066, UM111, UM352	
alpha-(1,3)-fucosyltransferase	WP_015646026.1	0	6 (50.0%)	10 (83.3%)	0.001*
			UM065, UM408, UM023, UM520, UM370, UM291	UM122, UM087, J99, UM163, UM119, UM085, UM096, UM077, UM066, UM111	

* p-value<0.05 is considered statistically significant

## Discussion

The identification of a set of genes that partition the good biofilm forming *H*. *pylori* strains from their poor biofilm forming counterparts demonstrate that biofilm formation phenotype is influenced by multiple genetic factors of the bacteria. Motility and adhesion capabilities apparently are critical for the initiation of biofilm formation (and probably the subsequent propagation of biofilm).

The movement ability by different species of bacteria is linked to their ability to colonize various ecological niches, and is frequently related to pathogenesis and biofilm formation. The gene encoding for hypothetical protein K747_06625 is predicted to contain ParB-like and HNH nuclease domains. In *Actinomyces oris* K20, genes containing ParB-like nuclease domain has been shown to be involved in the formation of meshwork-like structures, which are found in some biofilm-producing bacteria [32]. In *P*. *aeruginosa*, *par* mutants have been observed to be impaired in motility suggesting a direct or indirect role of Par proteins in regulation of these processes [33]. ParA/ParB family of proteins together with the centromere-like DNA sequence *parS* is involved in chromosome and plasmid partitioning during bacterial cell division. In the absence of ParB, ParA is most probably in its dimeric state bound with ATP proficient to bind non-specifically to DNA [34]. Furthermore, *par* mutation also induced genes induced involve in c-di-GMP turnover and signaling [33]. Cyclic-di-GMP (cyclic diguanylate) is an important messenger ubiquitous in bacterial cells controlling various processes, e.g. switch between the motile planktonic and biofilm lifestyles of bacteria, virulence of animal and plant pathogens, antibiotic production, progression through the cell cycle and other cellular functions [34].

Another gene-of-interest encodes for a flagellar protein, which is also involved in bacterial motility. It is believed that the presence of flagella provide motility that aids kinetics of biofilm formation [35]. Motility provides by flagella enhance the recruitment of planktonic cells to the biofilm [36]. Besides, the adhesive properties of flagella proteins might also promote biofilm formation in *H*. *pylori*. This was supported by previous studies that demonstrate that *Campylobacter jejuni* flagella are needed for the initiation of biofilm formation by mediating adhesion on the surface [37]. In one study, microcolonies were formed on a coverslip with flagella forming bridges between organisms [38]. During initial reversible attachment, flagella permit individual planktonic bacteria to swim toward an appropriate biotic or abiotic surface. Subsequently, during irreversible attachment, these flagella are lost and adhesive organelles became elaborated [39].

Biofilm forms a protective layer for Gram-negative bacteria to prevent attack by host immune system against one of its cell wall component, lipopolysaccharide (LPS) [40]. *H*. *pylori* alpha-(1,3)-fucosyltransferase, one of the many genes that involved in *H*. *pylori* LPS synthesis [41], is also predicted to contain the biofilm formation protein (YliH/bssR) domain, which has been shown to be induced in *Escherichia coli* biofilms [42]. In addition, *H*. *pylori*, alpha-(1,3/4)-fucosyltransferase is required for fucosylation of both type I (Le^a^) and type II (Le^x^) Lewis antigens [43]. The adhesive properties of Lewis X antigen enable first colonist to adhere to the surface of the liquid through weak adhesion force in the formation of biofilm [44].

One of the proteins found to be prevalent with good biofilm forming strains encodes for a *cag* pathogenicity islands protein. CagA protein, encoded by *cag* pathogenicity islands, has been identified to be induced in *H*. *pylori* biofilms [45]. CagA, which serves as a virulent factor of *H*. *pylori*, is injected into the host through the type IV secretion system and affect the individual [46]. Type IV secretion system-mediated transfer is essential in direct cell-cell contact [47]. It is believed that this direct cell-cell contact can control the biofilm behavior in *H*. *pylori* [48]. Furthermore, CagE, another protein encoded by the *cag* pathogenicity islands, has also been reported to be involved in the *H*. *pylori* biofilm formation [15,48]. *cagE* encodes for a cytoplasmic ATPase to allow the translocation of CagA protein that induces the gastric epithelial cells to secrete interleukin-8 (IL-8) and activate many intracellular signaling pathway [25]. Thus, the *cag* pathogenicity island may play an important role in *H*. *pylori* biofilm formation. Besides its role in bacteria-host interaction, CagA and the *cag* pathogenicity island may also have a role to play in bacteria-bacteria interaction in *H*. *pylori* biofilm formation.

## Conclusion

Using a comparative genomics approach, data from this study suggest that biofilm formation in *H*. *pylori* might be influenced by multiples genes. Good and poor biofilm forming strain may differ genetically in terms of motility, adhesion and bacteria-bacteria interactions, which are important during the initiation of biofilm formation.
